# Recurrent Granulosa Cell Tumor in a Postmenopausal Woman: A Case Report and Literature Review

**DOI:** 10.7759/cureus.43368

**Published:** 2023-08-12

**Authors:** Tsering Dolkar, Aditya Keerthi Rayapureddy, Nevil Kadakia, Amulya Bellamkonda, Madhumati Kalavar

**Affiliations:** 1 Internal Medicine, One Brooklyn Health (OBH) Interfaith Medical Center, New York City, USA; 2 Hematology and Oncology, One Brooklyn Health (OBH) Interfaith Medical Center, New York City, USA

**Keywords:** granulosa cell tumor chemotherapy, ovarian cancer staging, granulosa cell tumor recurrence, hyperestrogenism, ovarian cancer, sex cord stromal tumor, adult granulosa cell tumor

## Abstract

Ovarian cancer is among the most common types of cancer suffered by the female population. As of United States Cancer Statistics (USCS) 2019, the National Cancer Institute reports the prevalence of ovarian cancer as 11.4 cases per every 100,000 each year. The highest prevalence is in the seventh decade of life. Of all the types, sex cord-stromal tumors (SCSTs) account for 5-8% of cases. They are a heterogeneous group of rare neoplasms originating from the ovarian matrix, and nearly 90% of the hormone-producing tumors are SCSTs. Hence, patients with SCSTs are known to present with excess estrogen and androgen signs and symptoms. Many SCSTs are known for their indolent course and tendency to affect the unilateral ovary. The prognosis of the malignancy depends on the subtype of SCST, the stage of the patient's disease, and age. Among all the types, 20-50% of the ovaries' granulosa cell tumors tend to recur decades after the initial presentation, and 70% of the recurrences end up with a very poor prognosis. This case will discuss a 68-year-old woman who presented with a recurrence of an adult granulosa cell tumor after 13 years in remission. The patient had been previously diagnosed with an adult granulosa cell tumor of the right ovary at age 55 and had undergone surgical resection along with chemotherapy.

## Introduction

Sex cord-stromal tumors (SCSTs) occur in all ages and account for 7% of all ovarian cancers [1]. Of the many types of SCSTs, granulosa cell tumors (GCTs) alone account for 2-3% of all ovarian cancers [2]. According to the 2020 histological classification by the WHO of ovarian tumors, GCTs are of two kinds, adult granulosa cell tumors (AGCTs) and juvenile granulosa cell tumors (JGCTs) [3]. The granulosa cell tumor's clinical presentation is age-dependent based on the effect of the excess estrogen circulating. Where JGCTs may result in isosexual precocious puberty, AGCTs may present with menstrual irregularities, postmenopausal bleeding, endometrial hyperplasia, and even endometrial cancer [1]. Nevertheless, not all patients present due to excess estrogen but because of symptoms of a mass-occupying lesion such as abdominal pain, distension, or a frank abdominal mass. AGCTs are nearly always unilateral and have a very indolent course. Due to the symptoms, they are usually caught when the tumor is still in its initial stage (Stage I) of its progression and carry a remarkable 10-year survival of almost 95%. The cells formed are believed to arise from those cells surrounding the germinal cells within ovarian follicles. A microscopic examination of the AGCT specimen shows predominantly granulosa cells with pale, grooved "coffee bean" nuclei. The characteristic feature is a rosette arrangement of cells around the eosinophilic fluid space called the Call-Exner body [3]. Contemporary management of most patients with GCTs is surgical resection, whereas systemic therapy is reserved for metastasis or recurrence. Total abdominal hysterectomy with bilateral salpingo-oophorectomy is usually a surgical management choice in those with a complete family or extra ovarian disease [4]. GCTs are infamous for their tendency to relapse decades after the initial presentation; 15-20% of Stage I tumors will eventually return, usually in the abdomen or pelvis [5]. It is difficult to know what prognostic values or features can increase the risk of relapse. Various factors shown to have prognostic significance include age, tumor size (>10-15 cm in diameter), postoperative residual tumor, rupture of the tumor, mitotic activity (>4 mitoses per 10-high-power fields), nuclear atypia, aneuploidy (in 5-20% GCTs), p53 overexpression, and stage of the disease [6]. Here we present a case of an AGCT recurring 13 years after the initial tumor.

## Case presentation

**Figure 1:**
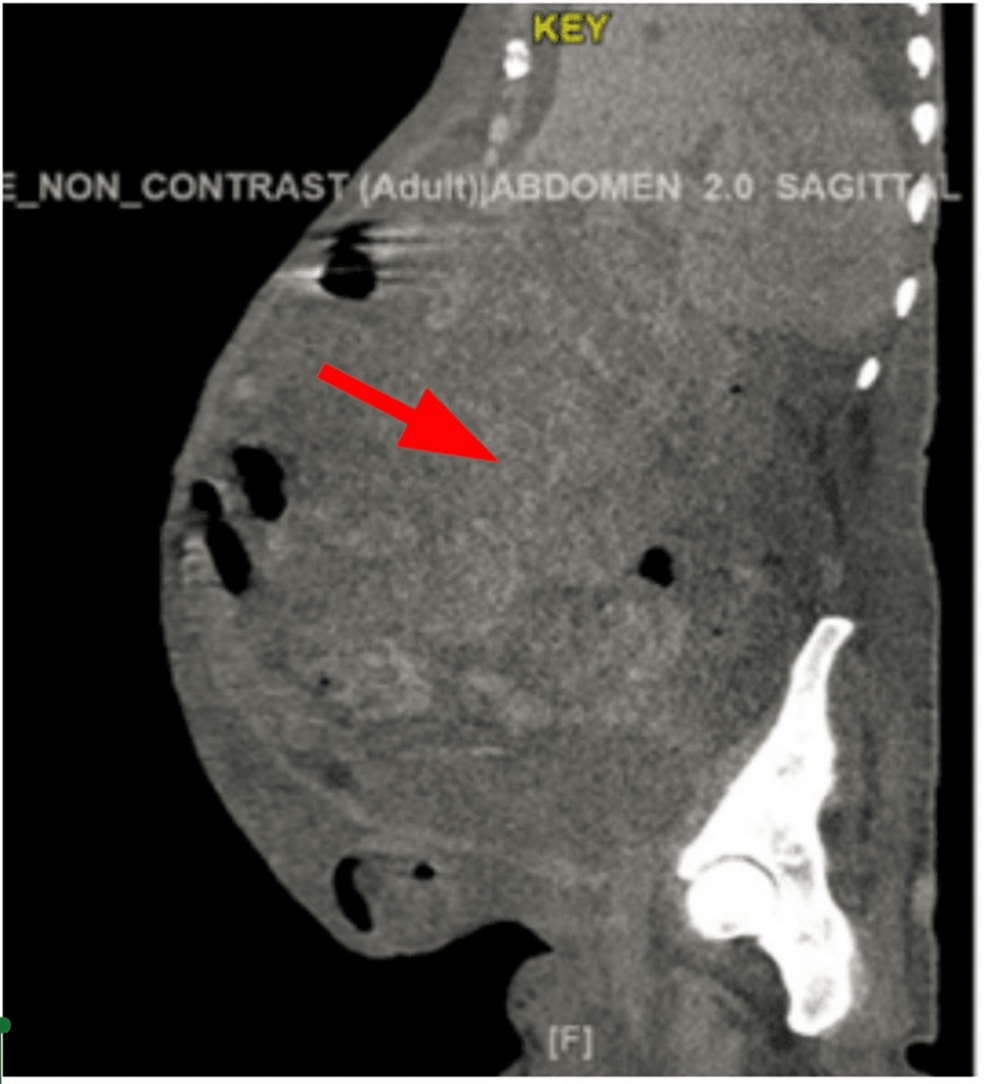
CT abdomen sagittal section: 20 x 20 x 30 cm abdominopelvic mass.

**Figure 2:**
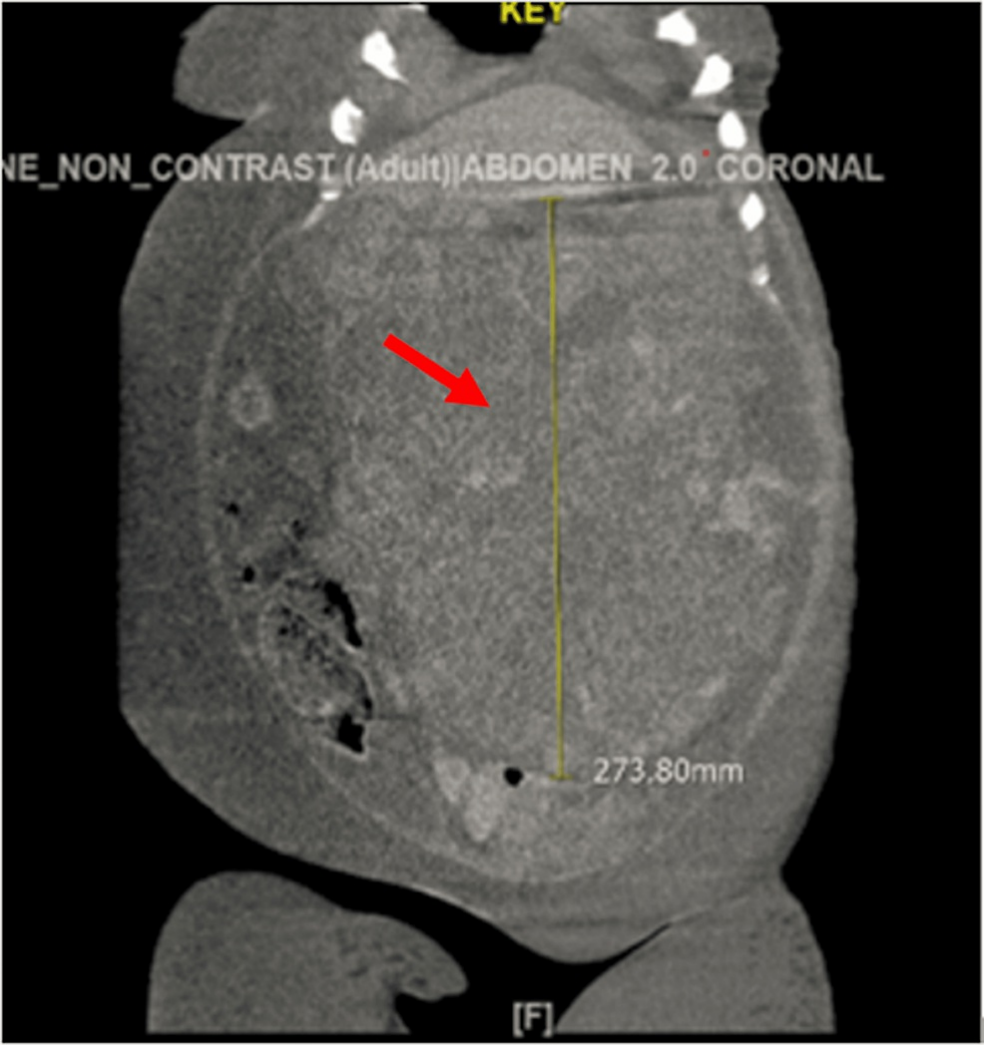
CT abdomen coronal section shows a huge abdominopelvic mass.

In 2018, at the age of 68 years, the patient presented to the oncology clinic with complaints of gradually progressive abdominal distension for a few months. The physical exam was unremarkable except for the abdominal distension. Vitals were unremarkable. She had undergone an evaluation for a hernia and was found to have a mass in her abdomen on CT abdomen (Figures 1-2).

**Figure 3:**
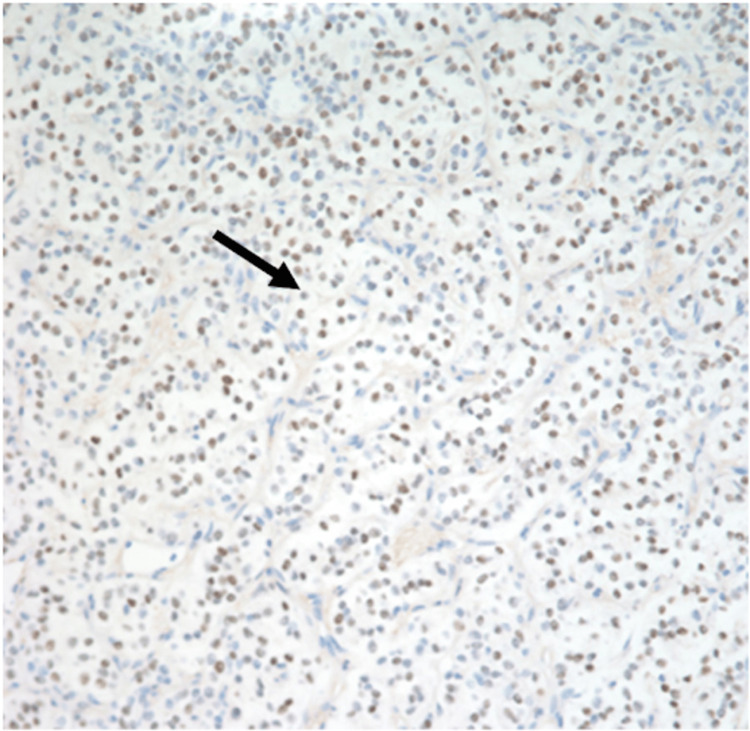
Immunohistochemical staining showing estrogen receptor (ER) positivity of tumor cells.

**Figure 4:**
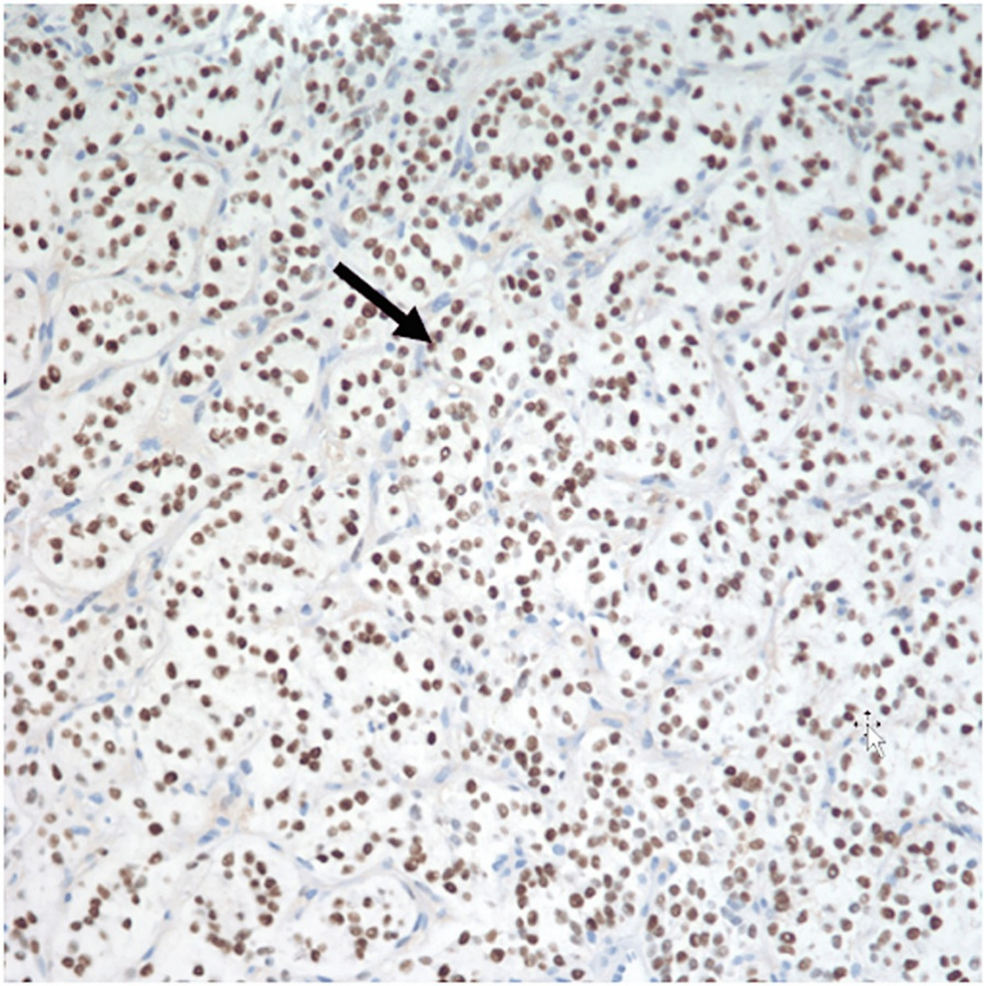
Immunohistochemical staining showing progesterone receptor (PR) positivity of tumor cells.

**Figure 5:**
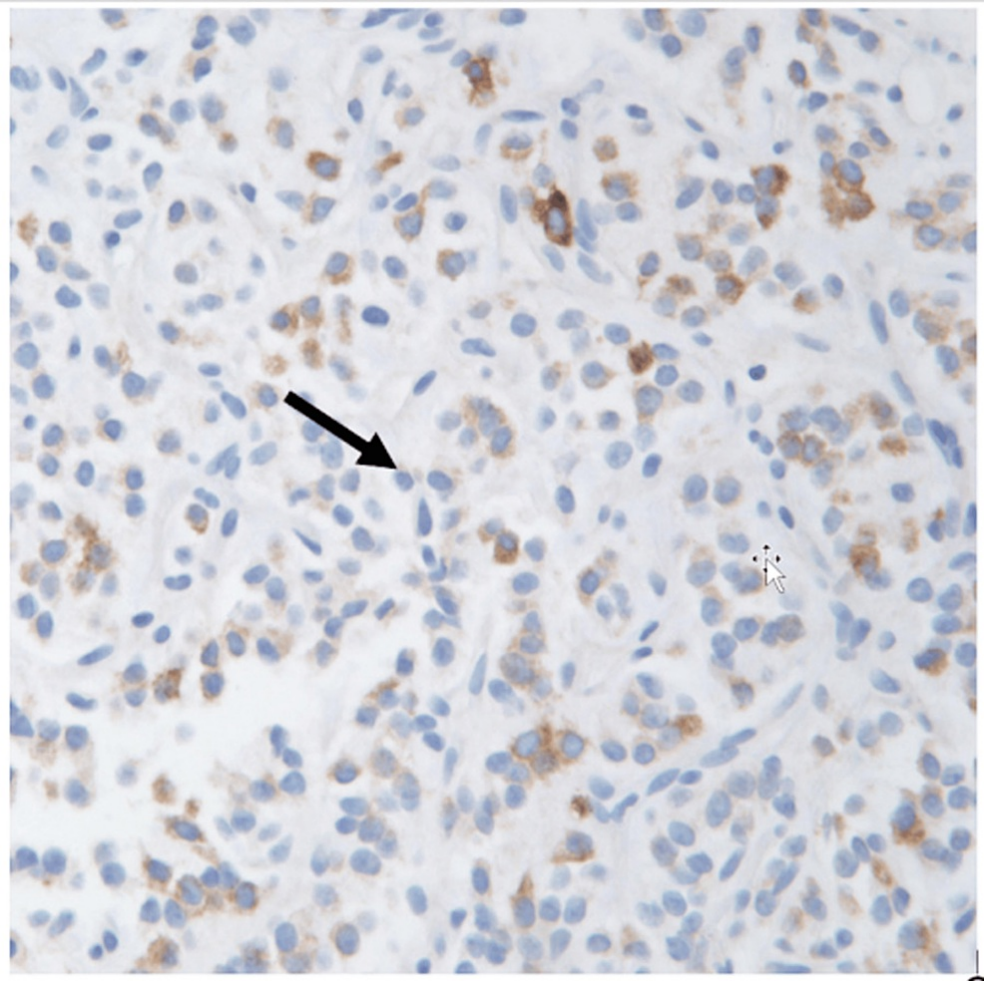
Immunohistochemical staining for inhibin showing cytoplasmic positivity in tumor cells.

A CT-guided core biopsy of the omental mass was performed, which demonstrated the presence of immunomarkers similar to those found in the GCT. The biopsy findings concluded with a diagnosis consistent with SCST of ovarian origin (Figures 3-5), which was ER+/PR+/S100 focally positive, inhibin focally positive, calretinin positive, and CD56 positive.

The patient had previously undergone a total abdominal hysterectomy with bilateral salpingo-oophorectomy (TAH BSO) after the tissue diagnosis of GCT Stage 1 in 2004. As per the pathology report, gross examination of the excised right adnexal mass revealed a partially encapsulated mass measuring 18 x 11 cm with shaggy, yellowish, brown, and hemorrhagic appearing non-encapsulated torn surfaces. Surgical staging and pathology report of the specimens obtained reported Stage 1C GCT of the right ovary. The patient had received three chemotherapy cycles with bleomycin, etoposide, and cisplatin (BEP). This latest omental mass is thus considered a recurrence of the previous tumor of ovarian origin. The patient had begun chemotherapy treatment with etoposide and cisplatin for the recurrent, now metastasized tumor. After the first cycle, the patient experienced clinical complications that resulted in hospitalization due to sepsis and had undergone a few months without chemotherapeutic treatment. On resumption of chemotherapy, cisplatin was discontinued due to patients' renal insufficiency. The chemotherapy regimen was adjusted to carboplatin and etoposide. The patient's clinical course was complicated by multiple hospital admissions for sepsis, severe anemia that required transfusions, chronic renal failure, and deep vein thrombosis. During one such hospital admission in 2019 for sepsis, the patient had to be discharged to a nursing facility for tracheostomy and percutaneous endoscopic gastrostomy (PEG). In 2022, she expired after contracting a COVID-19 infection. 

## Discussion

AGCTs are known for their tendency to recur decades after their initial presentation. Though GCT can recur in any region of the body, the pelvis and abdomen are observed to be the most common sites [5]. Here, we have a case of a woman diagnosed with an AGCT of the ovary initially at the age of 55 years, for which she had undergone a TAH BSO followed by three cytotoxic chemotherapy cycles with bleomycin, etoposide, and cisplatin. Post-treatment, the patient remained disease-free for 13 years until the age of 68, when she was diagnosed with a recurrence of the AGCT with the seeding to the omentum. Surgery is the cornerstone for definitive diagnosis, tumor debulking, and staging if imaging suggests malignancy in any patient with an ovarian mass without extra-abdominal metastasis. Since 2014, a common surgical staging system has been used for ovarian carcinoma, fallopian tubes, and peritoneum. It pertains to the staging of ovarian SCSTs as well. This common surgical staging has been designated by the International Federation of Gynecology and Obstetrics (FIGO) [7]. Surgical staging usually involves exploratory laparotomy with inspection and palpation of abdominopelvic contents, peritoneal washings or brushing for cytology, biopsy of any suspicious tissue, omentectomy, and bilateral pelvic and para-aortic lymphadenectomy for any lymphatic spread. However, GCT, like any other ovarian stromal tumor, has rare nodal involvement. Ovarian stromal tumors have a tendency for surface or hematogenous spread rather than a lymphatic spread [8]. For this reason, it has been observed that routine lymphadenectomy does not add much to the management of ovarian SCSTs, and bilateral pelvic and para-aortic lymphadenectomy can be safely omitted from the surgical staging of ovarian stromal tumors [9]. The surgical staging helps to identify the patients who would benefit from an adjuvant systemic therapy, usually reserved for metastatic or recurrent disease [4]. Most patients are diagnosed at an early stage, i.e., stage Ia. Sometimes, surgery alone should suffice with stage Ia tumors [9]. Patients presenting with stages Ic and higher, large tumor size (≥10-15 cm), poorly differentiated histology, and high mitotic index should be considered for adjuvant chemotherapy in view of the increased risk of relapse [10]. The lower the stage of the initial tumor at the time of resection, the better the prognosis of the recurrence [5]. A third of GCTs relapse between 4 and 7 years, leading to death in half of those recurrences [11]. A study by Cui X et al., which included 40 patients with recurrence of AGCTs, demonstrated a median overall survival rate of 126 months with a post-recurrence three-year survival rate of 82.4% and a five-year survival rate of 76.6%. This study also demonstrated that 20-25% of patients had a median recurrence time of 4-6 years after the treatment of the first recurrence [12]. Timely diagnosed patients with GCTs have a great outcome after undergoing appropriate treatment, with a median five and 10-year survival rate of nearly 95%, with a prognosis being related to the initial stage, presence of a residual tumor, and tumor size [13]. It has also been shown that younger age and early-stage disease are essential predictors for improved survival in patients with ovarian SCSTs [14]. The longest observed time interval between the initial presentation and recurrence is 37 years when the patient was 74 years of age, as reported in a case reported in 1995 [15]. AGCTs can show histo-morphological patterns similar to other unrelated tumors, so the diagnosis can be challenging. However, a single somatic missense mutation (C134W) in the gene encoding for transcription factor FOXL2 gene mutation (402C>G) is found to present in ~97%, i.e., most of the AGCTs. It is found in no other ovarian tumors, making it a characteristic feature specific to AGCT. This oncogenic mutation hinders the apoptosis of granulosa cells [16]. Detection of this FOXL2 mutation in ovarian-origin tumors can be useful in establishing an accurate diagnosis of AGCTs, as it is exclusive to the adult variant of GCT [11]. Tumor markers for a standard follow-up scheme are valuable. Serum markers, such as Inhibin B, are shown in some studies to be detected months before the recurrence and clinical detection. Currently, serum inhibin B has the most substantial evidence as a tumor marker for AGCTs, with a reported sensitivity between 88% and 100%. Anti-Mullerian hormone (AMH), a key regulator of follicular development in the ovary, has also been validated as a marker for AGCT. Furthermore, both AMH and Inhibin B are equally sensitive (92% and 93%) and specific for AGCT (82% and 83%) [11]. The combination of AMH and Inhibin B can be superior to Inhibin B alone in evaluating relapsed disease. It is recommended that both should be measured at the diagnosis and used for monitoring during the follow-up [11]. As stated earlier, surgery is mandatory for diagnosing, debulking, and initially managing a suspected GCT. Although TAH-BSO is the preferred surgical modality, women who want to preserve fertility can get a unilateral salpingo-oophorectomy if the tumor is confined to only one of the ovaries [9]. Stages Ic and above on surgical staging call for adjuvant systemic therapy. The most commonly used systemic therapy treatment is BEP [5, 17]. Efforts have been made to compare BEP therapy's efficacy and side effects vs. taxane-based therapy. One such retrospective study by Brown J et al. has shown that patients receiving taxane-based treatment for SCST showed no significant difference in response rate or progression-free survival, but it was less toxic than BEP [18]. The addition of bevacizumab to paclitaxel has not shown a clinical benefit either. Currently, the only efficacious targeted therapy in advanced ovarian cancer is bevacizumab, which works by inhibiting angiogenesis. The role of bevacizumab in recurrent AGCTs is underway in phase II clinical trials [11]. Hormonal therapy is reserved for patients who either did not respond to or could not tolerate conventional chemotherapy [4]. The treatment modalities of hormonal therapy have included progestins, gonadotropin-releasing hormone agonists, selective estrogen receptor modulators, and aromatase inhibitors [4]. Due to conflicting outcomes and a lack of randomized trials regarding hormonal therapies, more data are needed for objective analysis to establish their efficacy [11]. An in vitro study by Roze J et al. [19] tested all the current mono and combination therapies along with novel combinations on patient-derived AGCT cell lines. The cell lines were screened for drug responses. This study showed a consistent and synergistic inhibitory effect of PI3K inhibitor Alpelisib when used in conjunction with the current combination chemotherapy of paclitaxel and carboplatin. This is the only combination therapy of this study that showed a significant effect in 10 of the 12 (83%) tested patient-derived AGCT cell lines even after significant dose reductions, thereby suggesting a possibility for lesser toxicity. However, further in vivo studies are needed. This study also showcases that rapid, systemic, and patient-specific drug screens to test the sensitivity of drugs might be possible with patient-derived AGCT cell lines and to test for novel therapies. This might help with tailoring an efficient customized therapy for individual patients with a relatively lesser adverse side effect profile [19].

## Conclusions

Granulosa cell tumors are rare neoplasms with an indolent behavior. The optimal management of these tumors has never been determined by randomized trials. Although multiple studies have shown the efficacy of platinum-based regimens, there is a paucity of investigations and hence definite guidelines for angiogenesis inhibitors, hormonal therapy and radiation therapy. A recurrence with metastasis of granulosa cell tumor after 13 years, despite total hysterectomy with bilateral salpingo-oophorectomy and three cycles of chemotherapy clearly demands the need for increased post-treatment surveillance.
